# Selenium in Gluten-free Products

**DOI:** 10.1007/s11130-015-0467-8

**Published:** 2015-02-18

**Authors:** Iga Rybicka, Magdalena Krawczyk, Ewa Stanisz, Anna Gliszczyńska-Świgło

**Affiliations:** 1 Faculty of Commodity Science, Poznan University of Economics, al. Niepodległości 10, 61-875 Poznań, Poland; 2 Faculty of Chemical Technology, Poznan University of Technology, ul. Berdychowo 4, 60-965 Poznań, Poland

**Keywords:** Selenium, Gluten, Gluten-free diet, Celiac disease, Grain

## Abstract

The nutritional value of gluten-free products is the subject of interest for food technologists and nutritionists, as the only effective treatment for celiac disease is a lifelong gluten-free diet. As selenium deficiencies in celiac disease are observed, the aim of the study was to determine the selenium content in 27 grain gluten-free products available on the European Union (EU) market. Moreover, selenium content in products based on popular gluten-free cereals like corn, rice, and buckwheat and in relatively new or less popular products based on oat, amaranth, teff, and quinoa was compared. Selenium content in the tested products ranged from 0.9 to 24.5 μg/100 g. The average content of selenium in products based on popular gluten-free cereals was 2.8 μg/100 g and in products based on oat, amaranth, teff, and quinoa was 10.8 μg/100 g. It indicates that products based on less popular grains, especially on oat, should be more frequently chosen as a source of selenium by people on gluten-free diet than traditionally consumed gluten-free grains.

## Introduction

Gluten-free diet is proposed as a treatment of several illnesses [1], from which celiac disease (CD) is the most significant, as the restrictive menu without gluten is the only effective therapy for this affliction. Although it is estimated that 1–2 % of worldwide population suffers from intolerance to gluten, the real size of the problem is much bigger. It is associated with various, difficult to diagnose, clinical presentations of CD, among which the unique ones refer only to 10–20 % of patients [2]. Gluten-free diet has also become a new eating habit for healthy people who want to lose weight [3]. This interest reflects the annual increase in the market value of products with a Crossed Grain symbol, which is the fastest developing category of health and wellness products. It is accounted for 27 % of the whole food intolerance category [4]. Global value sales of gluten-free food reached US$ 2.1 billion in 2013 with annual growth up to 20 % since 2009 [5]. Currently, not only products from popular gluten-free cereals such as corn, rice, and buckwheat, but also from relatively new or less popular on the gluten-free market crops like oat, amaranth, teff, and quinoa are available. Moreover, the assortment of gluten-free products is expanding every year.

Grain products with a symbol of Crossed Grain are the only permitted on a gluten-free diet. Gluten in these products is naturally absent or technologically limited to the level below 20 mg/kg [6]. Gluten prolamins: gliadin, secalin, and hordein present in wheat, rye, and barley, respectively, need to be completely eliminated from the diet of CD patients. In this group, there is also oat contaminated with gluten grains, whose safety of use in gluten-free diet was widely discussed [7, 8]. In the Nordic countries, oat has been consumed by patients for several years, but *e.g*., in Poland the oat based products with a Crossed Grain symbol has become available for patients since 2013.

Selenium is present in the human body at a trace level. The largest quantities are cumulated in muscles, liver, kidney, pituitary, and thyroid gland. It occurs mainly as selenomethionine and selenocysteine. This element is a component of approximately 20 enzymes, including glutathione peroxidase that protects the organism from oxidative damage. As a part of selenoproteins and selenoenzymes it is considered a strong antioxidant, which together with antioxidant vitamins plays a protective role against the harmful effects of free radicals [9]. Selenium is essential for the proper synthesis, activation, and metabolism of thyroid hormones. It plays a role in the function of the immune system. Some studies report that sufficiently high intake of selenium may reduce the risk of certain cancers [10, 11] and have positive role in the prevention of inflammatory, cardiovascular, and neurological diseases [12]. Furthermore, selenium reduces the toxic effects of some xenobiotics, especially heavy metals, converting them to stable, less toxic forms. There is only a small difference between the recommended (55 μg) and the toxic dose (>400 μg) of selenium [9, 13]. Therefore, deficiency and redundancy of selenium are both dangerous for the human body [9].

The content of selenium in food is varied. Due to the fact that selenium is present in organisms in combination with proteins, products containing large amount of proteins are typically characterized by a high content of selenium. These products include: offal, meat and meat products, fish, seafood, dairy, nuts, and bread. Fruits and vegetables generally contain smaller amount of selenium, except for garlic, dry legumes, and mushrooms [14]. As carbohydrates should provide up to 65 % of daily requirements for energy, grain products are important nutritional components of a balanced diet. Properties and nutrient composition of different gluten-free products are described in the literature [15–19], but the data on selenium content refer mostly to gluten-free crops [20, 21]. To the best of the authors’ knowledge, only several gluten-free products are described in the literature in the aspect of selenium content [21, 22]. The USDA National Nutrient Database [21] does not contain the data for selenium content in crops like amaranth, teff, and quinoa, which are relatively new on the gluten-free market. Because of nutritional deficiencies observed in celiac patients [23, 24] and insufficient data on selenium content in food, the aspect of nutritional value of gluten-free products is indisputably important.

The aim of the study was to determine the selenium content in a range of grain gluten-free products. It is the first publication describing the content of this element in food products for people on a gluten-free diet. Moreover, the content of selenium in products from popular gluten-free cereals (corn, rice, buckwheat) and in relatively new or less popular on the market crops such as oat, amaranth, teff, and quinoa was compared.

## Materials and Methods

### Materials

Twenty seven gluten-free grain products: flours, breads, mixes for baking, pasta, flakes, snacks, and other were purchased in Polish health food stores, but they are mostly available throughout EU. All products were marked with a Crossed Grain symbol. Half of selected products (corn, rice, buckwheat) are commonly available on the market of gluten-free foods and are often consumed by celiac patients and people on a gluten-free diet. Other products (oat, amaranth, teff, quinoa) are relatively new on the market of food for particular nutritional uses and are rarely used in gluten free-diet.

Samples of products were prepared according to the similar procedure: ground in an agate mortar and sieved through a <2 mm sieve before analysis.

Accuracy of the analytical procedure described in this study was verified using Certified Reference Materials (CRMs): SRM 1567a (Wheat flour) and SRM 1549 (Non-fat milk powder) supplied by the National Institute of Standards and Technology (Gaithersburg, USA). All solid reference materials were used as bottled, without further grinding and sieving.

### Reagents and Gases

Working standard solutions were obtained by dilution of the stock standard solution (1000 mg/L solution of selenium in 0.5 mol/L nitric acid (Merck, Darmstadt, Germany)). Working standard solutions were prepared daily in high-purity deionized and double distilled water (quartz apparatus, Bi18, Heraeus, Hanau, Germany). Palladium modifier stock solution (10.0 ± 0.2 g/L) was obtained from Merck. 30 % H_2_O_2_ and 65 % HNO_3_ of the highest quality (Suprapur, Merck) were used for digestion of CRMs and real samples. Compressed argon of UHP 5.5 purity obtained from Air Products (Warsaw, Poland) was employed as a protective and purge gas.

### Instrumentation

A UniClever focused microwave sample preparation system (Plazmatronika, Wrocław, Poland) operating at 2450 MHz and 300 W maximum output was used for digestion of CRMs and real samples. The system with continuous temperature, pressure and microwave power monitoring was equipped with high-pressure TFM-PTFE vessel and water cooling system. The vessel capacity was 110 mL and the maximum pressure and maximum temperature were 100 atm and 300 °C, respectively. An Analytik Jena ContrAA 700 high-resolution atomic absorption spectrometer equipped with a 300 W xenon short-arc lamp (Analytik Jena, Jena, Germany) as a continuum radiation source was applied. A graphite furnace was used for atomization of analyte. The operating parameters of the HR-CS GFAAS (high-resolution continuum source graphite furnace atomic absorption spectrometry) instrument for selenium determination are summarized in Table 1.

**Table 1:** Operating conditions of the HR-CS GFAAS for selenium determination in food samples

AAS parameters	Values
Wavelength [nm]	196.0267
Lamp current [A]	9
Spectral range [pixel]	200
Dispersion [pm/pixel]	2
Read time [s]	5
Delay time [s]	0
Measurement mode	Peak height
Modifier	Pd(NO_3_)_2_
Modifier concentration [mg/L]	2
Modifier volume [μL]	5
Sample volume [μL]	30
Furnace program steps	
Drying	80 °C, ramp 6 °C/s, hold 20 s
Drying	90 °C, ramp 3 °C/s, hold 20 s
Drying	110 °C, ramp 5 °C/s, hold 10 s
Pyrolysis	350 °C, ramp 50 °C/s, hold 20 s
Pyrolysis	1050 °C, ramp 300 °C/s, hold 10 s
Gas adoption	1050 °C, ramp 0 °C/s, hold 5 s
Atomization	2100 °C, ramp 1500 °C/s, hold 4 s
Cleanout	2450 °C, ramp 500 °C/s, hold 4 s

### Microwave-Assisted Digestion of CRMs and Real Food Samples

The microwave-assisted digestion procedures for determination of metals in food samples can be found in the literature [25, 26]. Approximately 500 mg of powdered CRM/ground real sample was placed in the TFM-PTFE vessel of the microwave digestion system and moistened by 1 mL of 30 % H_2_O_2_. Then, 5 mL of 65 % HNO_3_ was added. The sample was heated for 20 min at 300 W. After mineralization, the clear digested solution was transferred into 10 mL calibrated flask and diluted to volume with high-purity water. A corresponding blank was also prepared according to the above microwave-assisted digestion procedure.

### HR-CS GFAAS Determination Procedure

The temperature program and the modifier used for selenium determination are shown in Table 1. The transient absorbance signals were integrated and both peak height and peak area signals were recorded. Peak height absorbance signals were used for calculations. Analytical blank was also carried through the whole procedure to correct possible contamination from the reagents used for the sample preparation. Quantification of selenium was done using external aqueous standard calibration curves. The limit of detection (LOD) calculated using the IUPAC recommendation (based on a 3σ_blank_ criterion) was obtained by the use of optimized operating conditions. Seven determinations of the total procedure (reagent) blank solution were carried out and the relative standard deviation (RSD) of the background values for the raw data was calculated. The limit of detection was 0.1 μg/L. The precision of replicate determination was approximately 10 % RSD.

### Determination of Water Content

Water content was determined using oven drying method [27].

### Statistical Analysis

Data are presented as mean ± SD of six determinations. Analysis of variance was performed on the obtained data using Statistica 10.0 program (StatSoft, Inc.). Significance of differences between means was determined by least significant differences (LSD) at α = 0.05.

## Results and Discussion

### Accuracy Verification

To ensure the accuracy and precision of the method, two certified reference materials (SRM 1567a (Wheat Flour) and SRM 1549 (Non-fat milk powder)) were analyzed. These reference materials were chosen as they were the closest available to gluten-free food products and were certified for the analyte of interest. Conventional calibration was compared with the standard addition slopes to evaluate the matrix effects on the analytical signals in digested samples. No significant differences were found between the slopes obtained by both calibration procedures. Therefore, conventional external calibration curve was used. The results obtained by external calibration technique for digested samples were in good agreement with certified values for CRMs according to the *t*-test at a 95 % confidence level (Table 2).

**Table 2:** Determination of selenium (μg/g) in CRMs using HR-CS GFAAS technique

Sample	Certified value	Found	Value of *t*-test
SRM 1567a (Wheat flour)	1.1 ± 0.2^NS^	1.0 ± 0.2^NS^	1.32
SRM 1549 (Non-fat milk powder)	0.11 ± 0.01^NS^	0.12 ± 0.02^NS^	1.32

Significance of *t*-test (*n* = 7) at 95 % confidence level; *t*_critical_ = 2.447; NS: not significant values in the raw

### Selenium Content in Gluten-Free Products

Twenty seven gluten-free cereal products were grouped into seven categories: flours, breads, flakes, pasta, cookies, snacks, and other. The mean content of selenium in selected products is presented in Table 3. The content of selenium ranged from 0.9 μg/100 g (corn flakes) to 24.5 μg/100 g (amaranth popping) with a mean of 6.7 μg/100 g. In two products (biscuits and pretzels with salt), the content of selenium was below LOD.

**Table 3:** Selenium content in gluten-free products

Category	Product	Selenium	
		[μg/100 g]	[μg/100 g d. m.]
Flour	Amaranth flour (roasted)	4.7 ± 0.3^a^	4.7 ± 0.3^a^
	Buckwheat flour	2.9 ± 0.3	3.2 ± 0.4^b^
	Corn flour	2.4 ± 0.2^b,c^	2.7 ± 0.2^b^
	Gluten-free flour with oat	1.3 ± 0.1	1.4 ± 0.1^d^
	Gluten-free flour with teff	11.4 ± 0.3^d^	12.0 ± 0.3^e^
	Oat flour	22.2 ± 1.3	23.5 ± 1.4
	Rice flour	2.5 ± 0.2^b,c^	2.6 ± 0.3^b,c^
Bread	Bread mix	1.8 ± 0.1^e^	1.9 ± 0.2^f^
	Oat bread mix	11.3 ± 1.3^d^	12.4 ± 1.4^e^
	Wholegrain bread mix	1.9 ± 0.2^e,f^	2.1 ± 0.3^c,f^
Flakes	Corn flakes	0.9 ± 0.1^g^	1.0 ± 0.1^g^
	Multigrain flakes with amaranth	1.5 ± 0.1^h^	1.6 ± 0.1^d,f^
	Oat flakes (oatmeal)	15.3 ± 0.5	16.7 ± 0.6
	Oat musli with fruits	4.2 ± 0.6^a^	4.7 ± 0.7^a^
	Rice flakes	2.2 ± 0.1^b,f^	2.3 ± 0.1^c,b^
Pasta	Pasta	1.5 ± 0.1^h^	1.7 ± 0.1^f^
	Pasta with buckwheat flour	2.3 ± 0.1^b,c^	2.5 ± 0.2^b^
	Pasta with teff	8.5 ± 1.1^i^	9.0 ± 1.3^h^
Cookies	Biscuits	<LOD^*^	<LOD^*^
	Muffin mix	10.7 ± 0.4^j^	12.0 ± 0.5^e^
	Oat muffin mix	13.0 ± 0.9	13.5 ± 1.1
	Oat cookie mix	9.7 ± 1.3^i,j^	10.0 ± 1.5^h^
Snacks	Pretzels with salt	<LOD^*^	<LOD^*^
	Sticks with salt	1.0 ± 0.1^g^	1.0 ± 0.1^g^
Other	Amaranth popping	24.5 ± 1.1	26.2 ± 1.2
	Chicory coffee	3.5 ± 0.3	3.9 ± 0.4
	Quinoa leaven	6.6 ± 0.4	6.9 ± 0.4

*d. m*. dry matter, means with the same letter in the row are not significantly different at α = 0.05; ^*^ below LOD

The average content of selenium in a group of gluten-free flours was 6.8 μg/100 g. In most of flours (amaranth, buckwheat, corn, gluten-free with oat, and rice) the content of this element did not exceed 4.7 ± 0.3 μg/100 g. Only two products: flour with teff (11.4 ± 0.3 μg/100 g) and oat flour (22.4 ± 1.8 μg/100 g) were richer sources of selenium.

In a group of mixes for gluten-free bread, oat bread mix contained more selenium (11.3 ± 0.3 μg/100 g) than bread mix based on gluten-free wheat starch, corn starch and corn flour (1.8 ± 0.1 μg/100 g) or wholegrain bread mix from corn starch (1.9 ± 0.2 μg/100 g).

The content of selenium in four from five gluten-free flakes ranged from 0.9 to 4.2 μg in 100 g. Only oat flakes were richer in this element, providing 15.3 ± 0.5 μg of selenium in 100 g.

The average content of selenium in gluten-free pasta was 4.1 μg/100 g with a wide range from 1.5 ± 0.1 μg/100 g (gluten-free pasta from corn and rice flour) to 8.5 ± 1.1 μg/100 g (pasta with teff).

A group of gluten-free cookies, without biscuits with selenium content below LOD, contained on average 11.1 μg of selenium in 100 g with the highest content of the element in oat muffin mix (13.0 ± 0.9 μg/100 g). The content of selenium in one from two gluten-free snacks, pretzels with salt, was below LOD and in sticks with salt was 1.0 ± 0.1 μg/100 g.

From other gluten-free products selected for the present study, the most valuable source of selenium was amaranth popping with the selenium content of 24.5 ± 1.3 μg in 100 g. Other products: chicory coffee and quinoa leaven contained 3.5 ± 0.3 μg and 6.6 ± 0.4 μg of selenium in 100 g, respectively.

### Comparison of Selenium Content Between Traditional and Less Popular Gluten-Free Products

**Fig. 1:**
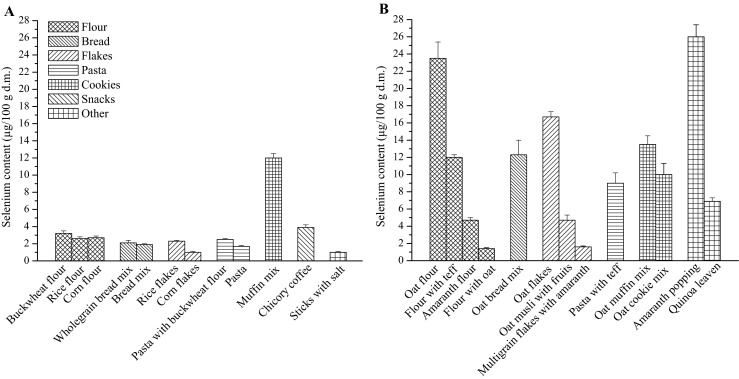
Comparison of selenium content in 100 g of dry matter of (**a**) products based on the most popular gluten-free cereals (corn, rice, buckwheat) and in (**b**) products based on less popular gluten-free cereals (oat, amaranth, teff, quinoa)

Twenty seven gluten-free grain products analyzed in the present study were grouped into two categories: (A) products from popular gluten-free cereals (corn, rice, buckwheat), and (B) products from less popular gluten-free crops (oat, amaranth, teff, and quinoa). Generally, the selenium content in a second group of products was higher than in the first one (Fig. 1). The average selenium content in A group was 2.8 μg/100 g and in B group was almost 4 times higher (10.8 μg/100 g).

It was found that relatively new on the market gluten-free flours from oat, amaranth, and teff may provide about 2–9 times more selenium than traditionally consumed flours from corn, rice, and buckwheat. From three mixes for bread, one was based on oat, two were from popular gluten-free grains. The first mix provided about 6 times more selenium than those based on corn or rice. The content of the element in oat flakes was especially high and it was about 7 and 17 times higher than in rice or corn flakes, respectively. Also, oat musli with fruits (dates, raisins, sunflower seeds, and apple) contained more selenium (4.2 μg/100 g) than popular gluten-free flakes (0.9 and 2.2 μg/100 g), however it should be mentioned that also fruits were the source of selenium in this product. Pasta with teff was about 4 and 6 times richer source of selenium than pasta with buckwheat flour or based on corn, respectively. Selenium content in traditional mix for gluten-free cookies based on corn starch and buckwheat flour (10.7 μg/100 g) and in cookies mixes from oat (on average 11.4 μg/100 g) was similar. A group of snacks and other gluten-free products differed in the level of selenium. Among snacks, traditional sticks with salt contained only 1.0 μg of selenium in 100 g, whereas selenium content in pretzels with salt was below of LOD. Amaranth popping was found to be rich in selenium. It may provide similar amount of this element as oat flour. Chicory coffee and quinoa leaven may provide more selenium than products based on popular gluten-free cereals (corn, rice, buckwheat) with an exception of muffin mix (Fig. 1). Such comparison of selenium content in traditional and relatively new gluten-free products is not available in the literature.

### Grain Gluten-free Products as a Source of Selenium in a Gluten-free Diet

The article presents the selenium content in a significant number of grain gluten-free products available on the EU market. Results of the present study are comparable to those found in the limited literature. Murphy and Cashaman [22] analyzed two gluten-free products and obtained the following results: 1.3 ± 0.3 μg of selenium in 100 g in gluten-free corn flour and 1.8 ± 0.3 μg/100 g in gluten-free bread. These results are comparable to the content of selenium in gluten-free corn flour (2.4 ± 0.2 μg/100 g) and gluten-free bread mix (1.8 ± 0.1 μg/100 g) of the present study. Only several traditional gluten-free products such as rice flour, corn flour, and corn flakes can be found in USDA National Nutrient Database, but none of the product is defined as “gluten-free” [21].

Gluten-free products based on corn, rice, and buckwheat are popular and often consumed by celiac patients [28]. Most of the flours, bread mixes, and other gluten-free products available on the worldwide market of food for particular uses are made from these grains. Oat, amaranth, teff, and quinoa products are not very popular among people on gluten-free diet, as they are relatively new on the market of gluten-free food. They are less accessible and more expensive than traditionally consumed grains. Moreover, oat was a subject of numerous studies on its safety on celiac diet [7, 8] and is available for patients only for several years or, in some countries, since 2013.

The richest sources of selenium are nuts, offal, meat, fish, seafood, eggs, and dairy products [14, 21]. Despite this, carbohydrates constitute the basis of a properly balanced diet, thus grain products should significantly realize the requirements for all nutrients, including selenium. It was found that from 27 selected products the most valuable source of selenium was oat products: flour, flakes, muffin and bread mix. Other important sources of selenium were amaranth popping, pasta with teff, and gluten-free flour with teff.

The paper indicates which grain gluten-free products are more valuable source of selenium and it may be a guidance on how to effectively compose the diet to achieve the optimal level of selenium and therefore how to avoid nutritional deficiencies. Milk with ready-to-eat flakes, which are often consumed for breakfast can constitute both a meal with relatively high or low selenium content. When a mix of oat flakes (30 g) and amaranth popping (10 g) is added to 300 mL of milk (2 % milk fat) [21], the content of selenium in meal could be about 14 μg. Selenium-poor flakes: corn flakes (30 g) and rice flakes (10 g) served with a portion (300 mL) of milk may provide almost two times less selenium (8 μg). In a balanced diet, breakfast should realize about 25 % of daily energy and nutrient requirements, thus the selenium-rich menu fully realize this assumption.

## Conclusions

Gluten-free grain products analyzed in this study significantly varied in the content of selenium. The obtained results indicate that the highest content of this element was in amaranth popping, oat flour, oat flakes, oat muffin mix, flour with teff, oat bread mix, and muffin mix. The content of selenium in these products was higher than 10 μg/100 g. Generally, relatively new or less popular gluten-free products from oat, amaranth, teff, and quinoa were more valuable source of selenium than the products based on traditional often-consumed gluten-free cereals: corn, rice, and buckwheat. From a nutritional point of view, products based, especially on oat, should be more frequently chosen by people on gluten-free diet than traditionally consumed grains.

## Abbreviations

CCD: Charge-coupled device
CD: Celiac disease
CRM: Certified Reference Material
EU: European Union
HR-CS GFAAS: High-resolution continuum source graphite furnace atomic absorption spectrometry
LOD: Limit of detection
